# Predictive Factors of Pulmonary Embolism in Older Patients with SARS-CoV-2: The OCTA-COVID-19 Study

**DOI:** 10.3390/jcm10132998

**Published:** 2021-07-05

**Authors:** Maribel Quezada-Feijoo, Mónica Ramos, Isabel Lozano-Montoya, Rocío Toro, Javier Jaramillo-Hídalgo, Eva Fernández de la Puente, Blanca Garmendia, Pamela Carrillo, Giovanna Cristofori, Saleta Goñi Rosón, Rocío Ayala, Mónica Sarro, Francisco J. Gómez-Pavón

**Affiliations:** 1 Cardiology Department, Hospital Central de la Cruz Roja, C/Reina Victoria, 24, 28003 Madrid, Spain; monica.ramos81@gmail.com (M.R.); rayalamunoz@gmail.com (R.A.); 2 School of Medicine, Alfonso X El Sabio University, Avda. de la Universidad, 1, Villanueva de la Callada, 28691 Madrid, Spain; isalozanomontoya@hotmail.com (I.L.-M.); jjaramillohidalgo@hotmail.es (J.J.-H.); blancagarmendia@gmail.com (B.G.); cpamela312@hotmail.com (P.C.); javiergomezpav@gmail.com (F.J.G.-P.); 3 Geriatric Department, Hospital Central de la Cruz Roja, C/Reina Victoria, 24, 28003 Madrid, Spain; evafdelapuente@hotmail.com (E.F.d.l.P.); giovanna.cristofori@gmail.com (G.C.); saleta.goni@gmail.com (S.G.R.); 4 Biomedical Research and Innovation Institute of Cadiz (INiBICA), Research Unit, Puerta del Mar University Hospital, Av/Ana de Viya 21, 11009 Cadiz, Spain; rociotorogreen@gmail.com; 5 Medicine Department, School of Medicine, Cádiz University, Edificio Andrés Segovia 3º Floor, C/Dr Marañón S/N, 21001 Cadiz, Spain; 6 Radiology Department, Hospital Central de La Cruz Roja, C/Reina Victoria, 24, 28003 Madrid, Spain; monicasarrorx@telefonica.net

**Keywords:** pulmonary embolism, older, COVID-19

## Abstract

Background: The risk of pulmonary embolism (PE) has not been studied in older patients affected by COVID-19. We aimed to assess PE incidence and risk factors in a population of older patients infected with SARS-CoV-2. Methods: An ambispective, observational cohort study. A total of 305 patients ≥ 75 years old had the SARS-CoV-2 infection from March to May 2020. The incidence rate of PE was estimated as the proportion of new cases within the whole sample. Youden’s index was used to assess the cutoff point of D-dimer. To select factors associated with the risk of PE, time-to-event analyses were performed using cause-specific hazard models. Results: In total, 305 patients with a median age of 87 years (62.3% female) were studied; 67.9% were referred from nursing homes and 90.4% received any type of anticoagulation. A total of 64.9% showed frailty and 44% presented with dementia. The PE incidence was 5.6%. The cutoff value of a D-dimer level over 2.59 mg/L showed a sensitivity of 82.4% and specificity of 73.8% in discriminating a PE diagnosis. In the multivariate analysis, the factors associated with PE were previous oncological events and D-dimer levels. Conclusions: The PE incidence was 5.6%, and major risk factors for PE were oncological antecedents and increased plasma D-dimer levels.

## 1. Introduction

Acute respiratory distress syndrome is related to high mortality among coronavirus disease 2019 (COVID-19) patients [1]. Recent observational studies have shown an increase in embolic events, including pulmonary embolism (PE). The incidence has been estimated to be 16 to 31% higher in critically ill patients than in those in a ward setting [2,3,4].

Elderly patients showed an increased prevalence of PE in the pre-COVID-19 era, which is partly due to comorbidities associated with PE that increase exponentially with age and are seven- to ten-fold more prevalent in patients of advanced aged than in younger patients [5,6]. Thus, PE exhibits a different incidence based on age and race. The population in the fifth decade accounts for 200 per 100,000 people, and that over 80 years old increases to 1134 per 100,000 [7,8,9]. The clinical features and outcomes of several cohorts have been published; however, age plays a significant role in the outcomes of these patients, and the data do not translate to older populations [10,11].

SARS-CoV-2 infection is related to coagulation impairment, with increased plasma levels of procoagulants, such as fibrinogen and D-dimer, and an increased mortality rate [12,13]. D-dimer is an accessible and reliable test for PE diagnosis, and its specificity decreases as age increases. In the elderly population, increased plasma concentrations have been proposed to rule out PE, thereby needing fewer diagnostic procedures [14]. D-dimer concentrations over 1.0 mg/L have been identified to have an unfavorable prognosis [15,16]. A D-dimer cutoff of 5.0 µg/mL was determined to be an independent predictor of PE in hospitalized COVID-19 patients [17].

PE risk in an elderly population severely affected by COVID-19 is not simply determined by the release of proinflammatory factors, such as IL-1 and Il-6, chemokines and the thrombotic response [18]. Several factors, such as male sex, oncological entities, hypoxemia, chronic obstructive pulmonary disease (COPD), pulmonary hypertension and heart failure (HF), also influence the severity of this entity. HF is a major risk factor for embolic events. Reduced mobility, venous stasis, blood viscosity and the proinflammatory state of HF facilitate PE [19,20]

Our objective was to demonstrate the incidence and risk factors of PE in an elderly cohort infected with SARS-CoV-2.

## 2. Material and Methods

### 2.1. Study Population

This ambispective, longitudinal, observational, cohort study was performed from March to May 2020. This study is part of the OCTA-COVID-19 study. Patients aged ≥75 years who were admitted to the Geriatric Medicine Department with a COVID-19 diagnosis were recruited. Patients were included if they tested positive for SARS-CoV-2 by reverse transcription polymerase chain reaction from a combined oropharyngeal swab with a clinical diagnosis of COVID-19. All clinical, laboratory and radiological data were collected from the medical report.

### 2.2. Ethics Approvals

The protocol was approved by the ethics committee under ID: I-4131. This study complied with the Declaration of Helsinki. All participants provided written informed consent. Collected data were appropriately made anonymous, and each patient was identified by a unique alphanumeric identification code.

### 2.3. Data Collection

The biodemographic data and clinical characteristics were collected at admission. The clinical variables included the presence of high blood pressure, diabetes mellitus type 2, atrial fibrillation (AF) and a history of congestive HF, PE, renal failure, COPD and cancer. The Clinical Frailty Scale (CFS) [21] was used to assess frailty in the two weeks before hospitalization. The degree of dependence was calculated using the Barthel Scale, [22] and the presence of dementia was assessed by the Global Deterioration Scale (GDS) [23].

### 2.4. Laboratory Procedures

SARS-CoV-2 detection was performed using real-time reverse transcription polymerase chain reaction (RT-PCR) on nasal swabs. Routine blood examinations included complete blood count, coagulation profile with D-dimer, serum biochemical tests (renal and liver function, creatine kinase, lactate dehydrogenase and electrolytes), serum ferritin and C-reactive protein (CRP). Chest radiographs were performed to assess the effects on the lungs.

### 2.5. Definitions

Patients were classified according to the clinical pretest probability as low, moderate and high risk according to the Wells scale, and the D-dimer levels were adjusted to the age of the patient, considering that the D-dimer level was elevated above 1 µg/mL. A positive CT scan confirmed the presence of PE, defined by the absence or presence of filling defects in one or more pulmonary arteries up to subsegmental arteries. Patients with a lower renal filtration <30 mL/mL/min/1.73 m^2^ were excluded from the CT scan. In those with filtration between 30–45 mL/min, a hydration protocol was carried out prior to the test.

Sepsis and septic shock were defined according to the 2016 Third International Consensus Definition for Sepsis and Septic Shock [24]. The pneumonia severity of the mortality scale CURB-65 was used, as recommended by the British Thoracic Society [25].

### 2.6. Outcomes

Our primary outcome was confirmed presence of PE. Secondary outcomes were determining risk factors for PE in an older cohort infected with SARS-CoV-2.

### 2.7. Statistical Analysis

Continuous variables are summarized as the medians and interquartile ranges (IQR), and categorical data are summarized as the frequencies and percentages. For univariate comparisons, the Mann–Whitney *U* test was used because of the nonnormal distribution of the continuous data. Categorical data were compared using the chi-square test or Fisher’s test, according to the expected counts.

The incidence rate of PE was estimated as the proportion of new cases within the whole sample, followed by 95% Wilson score confidence intervals.

The performance of the D-dimer level as a diagnostic predictor of PE was assessed. The area under the receiver operating characteristic (ROC) curve and a cutoff value were calculated using Youden’s index. Sensitivity, specificity, and predictive values and their 95% confidence limits were calculated.

To select factors associated with the risk of PE, time-to-event analyses were performed using cause-specific hazard models to account for the occurrence of death as a competing risk. Univariate analyses were carried out with selected variables according to the investigators’ criteria. Finally, statistically significant variables in the univariate analyses were included in a multivariate model.

All analyses were performed with SAS^®^ 9.4 (SAS Institute Inc., Cary, NC, USA). A *p*-value ≤ 0.05 was considered statistically significant.

## 3. Results

### 3.1. Participant Characteristics

In total, 305 patients (62.3% female) were included in this study (Table 1). The median age was 87 years old (IQR 82–91). A significant difference in age was observed between cohorts with and without PE involvement (*p* = 0.046). Detailed data regarding signs and symptoms, blood and radiological results and treatment are included.

Regarding the geriatric characteristics of this cohort, 207 (67.9%) patients were referred from a nursing home. A total of 205 (67.9%) subjects showed any kind of frailty or dementia (44.4%). Dementia was classified into Alzheimer’s in 69 patients (23.2%), vascular dementia in 23 (7.7%) and other types in 66 (22.1%). Of the patients with Alzheimer’s dementia, 17.4% had GDS4, 17.4% had GDS5, 44.9% had GDS6 and 15.9% had GDS7.

Regarding anticholinesterase treatment of all patients with Alzheimer’s dementia, 47.7% received some type of treatment. Forty-nine patients (16.1%) took some type of antipsychotic, of which 36 patients (11.8%) received quetiapine and 13 (4.3%) received risperidone.

The mean Charlson Comorbidity Index score was 2 (IQR 1–4), while the mean Barthel Index score was 65, which is compatible with a moderate degree of dependence. Among the geriatric syndromes, only frailty was highlighted; 35.3% of patients included in the PE cohort showed frailty compared to 69.8% in the non-PE cohort (*p* = 0.003).

Only previous oncological antecedents were significantly different between the groups (*p* = 0.0016). The median D-dimer level was higher in the PE group than in the non-PE group. However, the CRP level and ferritine level showed no differences between the groups. The mean hospitalization time in the PE cohort was longer than that in the non-PE group (24 days, (IQR 13–30) vs. 11 days, (IQR 6–17) (*p* = < 0.001)).

### 3.2. Incidence of Pulmonary Embolism

The presence of PE was observed in 17 of 305 patients, with an incidence of 5.6% (95% CI: 3.5% to 8.7%) diagnosed by positive computed tomography pulmonary angiography (CTPA). Fifteen patients (4.9%) had an isolated PE event, two patients (0.7%) were diagnosed with both PE and deep vein thrombosis and three patients (1%) had only deep vein thrombosis.

### 3.3. Sensitivity and Specificity of D-Dimer Measurement in the Geriatric Population with PE

**Figure 1:**
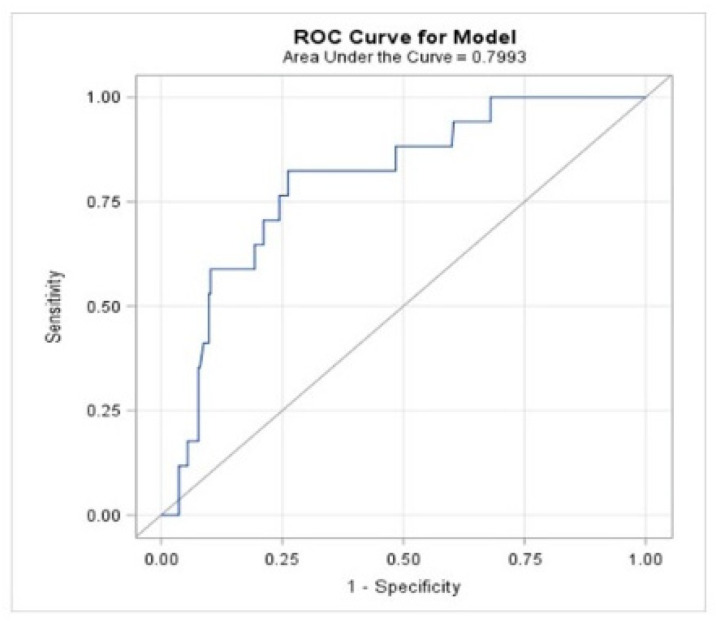
Receiver operating characteristic curve for D-dimer levels as a parameter for predicting pulmonary embolism in the COVID-19 cohort. The AUC for the model was 0.799.5% (0.699–0.899). The threshold for the D-dimer level was 2.59 µg/dL, which showed a sensitivity of 82.4% (59.0–93.8%), specificity of 73.8% (68.3–78.7%), positive predictive value of 16.3% (10.0–25.5%) and negative predictive value of 98.5% (95.8–99.5%).

We sought to identify the plasma D-dimer concentration value that could be considered a good and reliable discrimination marker of PE in our elderly population. The area under the curve (AUC) was lower than 0.799 (0.699–0.899) for a cutoff value of D-dimer concentration equal to or higher than 2.59 mg/L in patients over 70 years old (Figure 1).

We also classified patients according to whether their D-dimer was above the adjusted D-dimer level (that is, age divided by 100 mg/L units). Regarding this adjusted D-dimer, the results were: Sensitivity 100% (81.6–100), Specificity 22.9% (18.4–28.1), PPV 7.1% (4.5–11.1), 100% NPV (94.5–100).

### 3.4. Risk Factors Related to PE in Patients Affected by COVID-19

The variables associated with PE and mortality in the univariate analysis were age, previous oncological history, frailty, tachycardia and D-dimer level. Using hazard models, previous oncological events were a strong independent predictor of PE (HR 3.17; 95% CI 1.15–8.74) (*p* = 0.026)), followed by the D-dimer level (HR 1.02; 95% CI 0.999–1.049) (*p* = 0.062)). Nevertheless, frailty showed a protective effect, meaning that fewer patients with frailty showed PE (HR 0.34; 95% CI 0.999–1.049) (*p* = 0.054)) (Table 2).

## 4. Discussion

### Key Findings

PE is a dynamic process that shares common features with HF and COPD and makes the diagnosis complex. Thus, PE is over- and underdiagnosed. In the early stages of the pandemic, dyspnea, dementia, delirium and the excessive increase in D-dimer levels in the elderly population affected by COVID-19 led to confusion in the early diagnosis of PE, evolving therapeutic management from the adaptation stage to the arduous stage. To our knowledge, this is the oldest cohort affected by COVID-19. We studied 305 patients hospitalized in a secondary geriatric hospital with a PE incidence of 17/305 patients, which represents an incidence of 5.6% (95% CI: 3.5% to 8.7%). This incidence is higher than that reported by Lodiagini et al. [3] with 10/362 patients (2.8%) or Benito et al. [26], who described an incidence of 2.6% PE in 1275 inpatients who underwent CTPA. This could be explained by the scarcity of CTPA performed in the latter population, underestimating the true figure of affected patients.

The Prophylaxis in Medical Patients with Enoxaparin (MEDENOX) trial, which was performed in the pre-COVID-19 era on a population with acute medical illness with moderate risk of a thromboembolic event [27], set the embolic event reduction and beneficial anticoagulant prophylaxis for acute disease cohorts. Although most of the hospitalized patients with COVID-19 were on anticoagulant therapy, the incidence of PE was high. SARS-CoV-2 infection promotes endothelial dysfunction, prothrombotic events and pulmonary microthrombi, and the inflammatory host response leading to PE has been proven in autopsy studies [28]. Poissy et al. [29] showed that patients with COVID-19 infection had a higher frequency of PE than patients affected with other infections. They also proposed a pulmonary thrombosis mechanism rather than embolism due to the low diagnostic deep vein thromboses found.

The D-dimer level was increased in the PE cohort. This increased D-dimer concentration mirrors SARS-CoV-2 and acute lung injury, decreasing the PaO2/FiO2 ratio (PAFI) and driving coagulation activation secondary to the inflammatory response.

This threshold of the D-dimer concentration, 2.59 mg/L, is similar to that reported in other works. Leonard-Lorant et al. [30] established a cutoff point of 2.66 mg/L, allowing the detection of PE in all patients for whom CTPA was performed. On the other hand, Cui et al. [31] used a lower plasma level of D-dimer as a predictor of embolic events. They proposed increasing the threshold to 3 mg/L to detect the high-risk group for pulmonary events; this cutoff value improved the specificity to 94.9% and the negative predictive value to 92.5%. Unlike the aforementioned authors, our study population consisted of older adults, many of whom had frailty, some degree of dependence and other pathologies that may mask symptoms, leading to underprescription of the diagnostic test and possible misdiagnosis of PE. Thus, we propose this cutoff value for the D-dimer concentration in this population to support the detection of embolic events in elderly patients infected with SARS-CoV-2.

Several predictive factors of PE in elderly patients affected by COVID-19 have been described, such as unit care admission, the time of hospitalization and D-dimer levels [32,33]. We confirmed, according to Al-Samkari et al. [34], that a D-dimer level of 1.0 to 2.5 mg/L had an odds ratio (OR) for thrombotic complications of 3.04. In this sense, a plasma D-dimer plasma concentration over 1 µg/mL was associated with a risk of PE. Benito et al. [26] proposed that levels of CRP over 150 mg/dL and D-dimer over 1 mg/L could identify patients at risk for PE, who therefore could be candidates for increased thromboprophylaxis doses. CTPA should be performed when the D-dimer level persistently increases.

The predictive factors for PE found in our study help identify patients who will benefit from an early diagnosis of PE. This cohort is more vulnerable to several comorbidities and a longer in-hospital stay. According to a French multicenter cohort study, our mean in-hospital length of stay increased significantly in the PE group, from 11 to 24 days [35].

The wide clinical spectrum of PE related to oncological events has been established as an important predictor of PE in our population. Before the COVID-19 era, these data were confirmed, showing a four- to seven-fold increase in thrombotic complications in oncological patients compared with those in the general population [36]. Thus, the presence of oncological history was an independent mortality factor for elderly patients with PE events six months after hospital discharge [37]. Surprisingly, a traditional risk factor for PE as an oncological event has not been assessed in different COVID-19 patients [19,38]. Cancer was present in 18% of RIETE registry [39] patients over 80 years old with venous thrombosis, which was similar to the percentage of younger patients but higher than that observed in other COVID-19 series where the mean age was 65 years old [3,11]. This high oncological prevalence and its relation to thrombotic events could help identify patients at risk.

Al Ghatrif et al. [40] demonstrated that age could influence COVID-19 infection. Older patients have shown a reduced concentration of angiotensin-converting enzyme-2; moreover, oxidative stress and the inflammatory response have an increased intensity and could also be promoted by tumor progression.

Our results confirm that, within the senior population, there are different outcomes in younger patients than in the group over 85 years old. Patients younger than 85 years old with reduced frailty showed an increased risk of PE. Our study demonstrates an increased prevalence of frailty (67%) in patients admitted to the hospital due to COVID-19 compared to other series reported in the multicenter COVID-19 in Older People (COPE) study [41]. Our population was recruited from a geriatric hospital, and an increased number of participants came from nursing homes. To our knowledge, no study has related frailty to embolic events. The most interesting results in our cohort are that the less frail group showed more PE, confirming the association between inflammation and thrombosis [42]. This study reported that low-to-moderate fragility was associated with higher inflammatory markers than severe and very severe frailty. The inflammatory response is the basis of the defense against external and internal injuries [43]. In the young population, this response is needed against infectious diseases. The context is different in 90- and 100--year-old patients who may have retarded inflammatory biomarkers that delay age-related diseases and protect against the adverse effects of maintaining the response. In this context, immunosenescence could influence the response of the immune system to an acute infection, such as COVID-19. This response could limit the cytokine storm in geriatric patients and slow the prothrombotic and inflammatory cascades [44,45].

Although immunosenescence phenomena have gained interest in recent years, their molecular mechanisms have not been completely studied. Due to the drastic increase in the global life expectancy, more in-depth studies are required to identify and understand how aging influences the immune system. The clinical implications of these findings remain to be explored in larger studies with longitudinal follow-up. Furthermore, it would be valuable to compare patients with COVID-19 to patients who have experienced other viral infections.

## 5. Conclusions

The difficulty in the early diagnosis of PE in older patients infected by COVID-19 represents a challenge in the in-hospital management of these patients. The findings of this study can help understand the population at risk of developing PE and avoid unnecessary testing. The incidence of PE in the geriatric population tested by CTPA was 5.6%. Cancer and D-dimer concentrations were independent factors associated with PE in this population. The D-dimer cutoff value of 2.59 mg/L appears to be a good discriminator of PE, with a sensitivity of 82.4% and specificity of 73.8% for detecting new cases.

## 6. Limitations

This study has several limitations. First, due to the intrinsic characteristics of the pandemic and the high workload, there was a relatively short time to collect data during patient hospitalization. Second, there was a restriction on accessing different diagnostic tests and complex logistics to confirm PE, such as CTPA, the gold standard test. Third, the severity in our cohort at the very beginning of the pandemic could have influenced the diagnosis of PE. Finally, variables for the analysis were collected at admission; there was a lack of follow-up and the dynamic influence of COVID-19 disease evolution was not considered.

## Figures and Tables

**Table 1:** Characteristics of the population of the study.

	Global Population	PE (*n* = 17)	Non-PE (*n* = 288)	*p*
Age (years), median	87 (82–91)	83 (80–86)	87 (82–91)	0.046
Sex (male)	115 (37.7)	9 (52.9)	106 (36.8)	0.182
Place of origin				0.805
Home	98 (32.1)	5 (29.4)	93 (32.3)	
Nursing home	207 (67.9)	12 (70.6)	195 (67.7)	
Days since COVID-19 symptom onset	5 (2–7)	5 (2–10)	5 (3–7)	0.892
Comorbidities				
Arterial hypertension	204 (66.9)	9 (52.9)	195 (67.7)	0.209
Diabetes mellitus	86 (28.2)	5 (29.4)	81 (28.1)	1.000
Atrial fibrillation	85 (27.9)	4 (23.5)	81 (28.1)	0.788
COPD	48 (15.7)	3 (17.6)	45 (15.6)	0.737
Chronic renal dysfunction	53 (17.4)	0 (0)	53 (18.4)	0.051
Oncological history	41 (13.4)	6 (35.3)	35 (12.2)	0.016
Prior PE	18 (5.9)	1 (5.9)	17 (5.9)	1.000
Heart failure	74 (24.3)	2 (11.8)	72 (25.0)	0.380
Geriatrics assessment				
Charlson Comorbidity Index	2 (1–4)	3 (1–5)	2 (1–4)	0.272
Barthel Index categories	65 (29–90)	75 (60–100)	64.5 (26–90)	0.061
CFS	205 (67.9)	6 (35.3)	199 (69.8)	0.003
Global deterioration scale	134 (44.4)	6 (35.3)	128 (44.9)	0.438
Chronic treatment				
Angiotensin-converting enzyme inhibitors	150 (49.2)	9 (52.9)	141 (49.0)	0.750
Angiotensin receptor blockers	53 (17.4)	3 (17.6)	50 (17.4)	1.000
Oral antidiabetics	55 (18.0)	4 (23.5)	51 (17.7)	0.521
Oral anticoagulation	84 (27.5)	3 (17.6)	81 (28.1)	0.417
Beta-blockers	57 (18.7)	4 (23.5)	53 (18.4)	0.534
Digoxin	12 (3.9)	0 (0)	12 (4.2)	1.000
Antidepressants	111 (36.4)	6 (35.3)	105 (36.5)	0.923
Quetiapine	36 (11.8)	3 (17.6)	33 (11.5)	0.435
Symptoms at hospitalization				
Cough	136 (44.7)	5 (29.4)	131 (45.6)	0.191
Fever	156 (51.1)	8 (47.1)	148 (51.4)	0.729
Dyspnea	179 (58.7)	12 (70.6)	167 (58.0)	0.305
Falls	33 (10.8)	4 (23.5)	29 (10.1)	0.098
Delirium	112 (36.7)	5 (29.4)	107 (37.2)	0.520
Clinical signs				
Fever	36.9 (36.3–37.8)	37.05 (36.6–38.15)	36.9 (36.3–37.8)	0.400
Blood oxygen saturation (%)	92 (88–95)	92 (88–94)	93 (88–95)	0.285
Blood pressure (mmHg)	129 (110–147)	124 (110–138)	129 (110–148)	0.334
Respiratory rate (breaths/min)	20 (18–28)	21 (18–28)	20 (18–28)	0.793
Heart rate (beats/min)	84 (72–97)	95 (85–106)	83 (72–96)	0.007
CURB-65	2 (1–3)	2 (1–3)	2 (1–3)	0.518
SOFA	1 (0–1)	0 (0–1)	1 (0–1)	0.111
Blood test				
Platelets, median (10^3^/µL)	230 (167–324)	229 (208–248)	230 (164–329)	0.710
Leukocytes	7620 (5570–9970)	7200 (5920–8880)	7640 (5565–10,180)	0.483
Lymphocytes (10^3^/µL)	0.89 (0.6–1.25)	0.82 (0.47–1.1)	0.9 (0.61–1.27)	0.421
CRP mg/L	65 (26.9–151)	49 (21–158)	66.4 (27–149)	0.783
Creatinine (mg/dL)	0.97 (0.7–1.4)	1 (0.63–1.3)	0.97 (0.7–1.4)	0.739
Ferritin (ng/dL)	267 (156–469.5)	212 (178 – 586)	278 (156 – 467)	0.905
Peak D-dimer (mg/L)	1.66 (0.89–3.16)	6.14 (2.94–8.71)	1.58 (0.86–2.93)	<0.001
Radiology				
Pneumonia	250 (82.0)	11 (64.7)	239 (83.0)	0.095
Pneumonia type				0.227
Unilateral	25 (8.2)	0 (0)	25 (8.7)	
Bilateral	60 (19.7)	2 (11.8)	58 (20.1)	
Multilobar	165 (54.1)	9 (52.9)	156 (54.2)	
Medication				
Hydroxychloroquine	264 (86.6)	13 (76.5)	251 (87.2)	0.261
Azithromycin	191 (63.0)	11 (64.7)	180 (62.9)	0.883
Lopinavir	22 (7.2)	2 (11.8)	20 (6.9)	0.351
Steroids	111 (37.1)	7 (41.2)	104 (36.9)	0.722
Hospitalization				
Length of in-hospital stay (days)	11 (6–18)	24 (13–30)	11 (6–17)	<0.001
Discharge	193 (63.3)	14 (82.4)	179 (62.2)	0.093
Death, *n* (%)	112 (36.7)	3 (17.6)	109 (37.8)	0.093

CFS—Clinical Frailty Scale; COPD—chronic obstructive pulmonary disease; CURB-65—severity score for predicting mortality from community-acquired pneumonia. PE—pulmonary embolism; CRP—C-reactive protein; SOFA—Sequential Organ Failure Assessment. Data are presented as the medians and interquartile ranges or numbers (%).

**Table 2:** Univariate and multivariate analysis of our study population.

	HR (95% CI) Univariant	*p*	HR (95% CI) Multivariant	*p*
Age (years)	0.93 (0.87–0.99)	0.025		
Age ≥ 85	0.33 (0.12–0.89)	0.029	0.46 (0.15–1.39)	0.167
Diabetes mellitus	0.92 (0.34–2.52)	0.874		
Oncological history	3.44 (1.32–9.01)	0.012	3.17 (1.15–8.74)	0.026
Heart failure	0.37 (0.08–1.66)	0.194		
Prior PE	1.14 (0.16–8.14)	0.898		
Deep venous thrombosis	0.73 (0.11–4.93)	0.745		
Falls	2.29 (0.78–6.72)	0.131		
Dementia	0.66 (0.25–1.76)	0.403		
Acetylcholinesterase Inhibitors	2.94 (0.93–9.28	0.067		
Antipsychotic drugs	1.11 (0.31–3.96)	0.876		
Frailty	0.23 (0.09–0.60)	0.003	0.34 (0.11–1.02)	0.054
Dependence	0.24 (0.09–0.63)	0.004		
Dyspnea	1.55 (0.55–4.34)	0.408		
Respiratory rate (breaths/min)	1.00 (0.94–1.06)	0.961		
Tachycardia	1.02 (1.01–1.04)	0.001		
CRP mL/L	1.00 (0.994 – 1.004)	0.724		
Ferritine (ng/mL)	1.00 (0.999 – 1.001)	0.743		
D-dimer	1.03 (1.004–1.048)	0.020	1.02 (0.999–1.049)	0.062
D-dimer ≥ (2.59 mg/L)	10.06 (2.90–34.92)	<0.001		

PE—pulmonary embolism; HF—heart failure; CRP—C-reactive protein; HR—hazard rate, CI—confidence interval.

## Data Availability

The data that support the findings of this study are available on request from the corresponding author. The data are not publicly available due to privacy or ethical restrictions.
